# Muscle Strength and Physical Performance Are Associated with Reaction Time Performance in Older People

**DOI:** 10.3390/ijerph18115893

**Published:** 2021-05-31

**Authors:** José Daniel Jiménez-García, Antonio Martínez-Amat, Fidel Hita-Contreras, Raquel Fábrega-Cuadros, Francisco Álvarez-Salvago, Agustín Aibar-Almazán

**Affiliations:** 1 MOVE-IT Research Group and Department of Physical Education, Faculty of Education Sciences, University of Cádiz, 11003 Cádiz, Spain; josedanieljimenezgarcia@gmail.com; 2 Department of Health Sciences, Faculty of Health Sciences, University of Jaén, 23071 Jaén, Spain; amamat@ujaen.es (A.M.-A.); fhita@ujaen.es (F.H.-C.); aaibar@ujaen.es (A.A.-A.); 3 Department of Physiotherapy, Faculty of Health Sciences, European University of Valencia, 46112 Valencia, Spain; salvagofran@gmail.com

**Keywords:** reaction times, elderly, sarcopenia, physical fitness, physical function

## Abstract

Background: Functional mobility and muscle strength are well known risk factors for sarcopenia. Furthermore, possible associations have been suggested between predisposing factors of sarcopenia and reaction time among the elderly. This study aims to analyze possible associations of functional mobility and muscle strength and reaction times in a population of people aged >60 years. Methods: A total of 290 older people (69.35 ± 5.55 years) participated in this study. The following parameters were assessed: optoacoustic lower-limb reaction time (OALLRT); acoustic lower-limb reaction time (ALLRT); optic lower-limb reaction time (OLLRT, using an optical detection system), functional mobility (through the timed up-and-go test) and muscle strength (using a dynamometer). Results: Our results show that lower values of muscle strength were associated with increased reaction times in OALLRT (β = −0.170; 95% confidence interval −0.011–0.000; R^2^ = 0.237; *p* = 0.035) and in ALLRT (β = −0.228; 95% confidence interval −0.011–0.002; R^2^ = 0.199; *p* = 0.006). Conclusion: Increased muscle strength (which at low values are risk factors for sarcopenia) was associated with decreased reaction times in people >60 years of age.

## 1. Introduction

Sarcopenia is increasingly becoming a global health concern with multiple and far-reaching health-related implications, not only at the individual level but also for society at large. As a result, the concept appears with increasing frequency in clinical practice [1,2,3,4]. Nowadays, sarcopenia is a syndrome that affects over 50 million people around the world, a figure that is expected to triple in the next 20 years. Specifically, among older populations this syndrome has been demonstrated to affect 5–13% of persons aged 60 to 70 years and up to 50% of people over 80 years of age [5,6].

In 2010, The European Working Group on Sarcopenia in Older People (EWGSOP) established a definition for sarcopenia that would allow the development of new strategies for its treatment and management. In early 2018 the group decided to meet again (EWGSOP2) and update the original definition. EWGSOP2 states that low muscle strength (probable sarcopenia) should be measured by grip strength and/or the chair stand test, and how this is enough to search the causes and start an intervention, although the diagnosis must be confirmed by additional information such as low muscle quality. Furthermore, when low muscle strength, low muscle quality, and low functional mobility are all present, sarcopenia is deemed to be severe [7]. In light of this, being able to identify sarcopenic individuals may allow for a variety of therapeutic approaches to be implemented, aiming to prevent a decline in which falls [8], frailty syndrome [9,10], and other adverse health outcomes [11,12] become increasingly frequent.

The literature has identified that the presence of low muscle strength, low functional mobility, or low muscle mass, (or a combination thereof) is one of the most significant causes of functional decrease and loss of independence in older adults, due to its proven association with acute and chronic disease states, increased insulin resistance, fatigue, rheumatologic conditions, and mortality [8,13,14]. It has also been stated that, among this population, slow reaction times are associated with an increased risk of falling [8,14] and that protective responses tend to be ineffective because of the lack of strength and movement speed [15]. Therefore, and bearing in mind that the consequences of falls are known to bring about a plethora of health concerns and increased health-care cost for older adults [16], exploring whether and how muscle strength and functional mobility affect reaction times among the elderly becomes particularly relevant.

In that regard, several studies have employed a varied selection of tests for the quantification of muscle strength (e.g., handgrip strength and knee flexion/extension testing) [17,18], and the assessment of functional mobility (e.g., gait speed, the short physical performance battery (SPPB), the 6 min walk test, the timed up-and-go test (TUG), and the stair climb power test) [19,20,21]. Others, although only a few, have suggested possible associations between these predisposing factors and reaction times among elderly people [22], which highlights the need for more evidence in this field. For this reason, the purpose of this paper is to analyze the possible associations between both muscle strength and physical performance and several reaction times among this population.

## 2. Materials and Methods

### 2.1. Study Design and Participants

**Figure 1:**
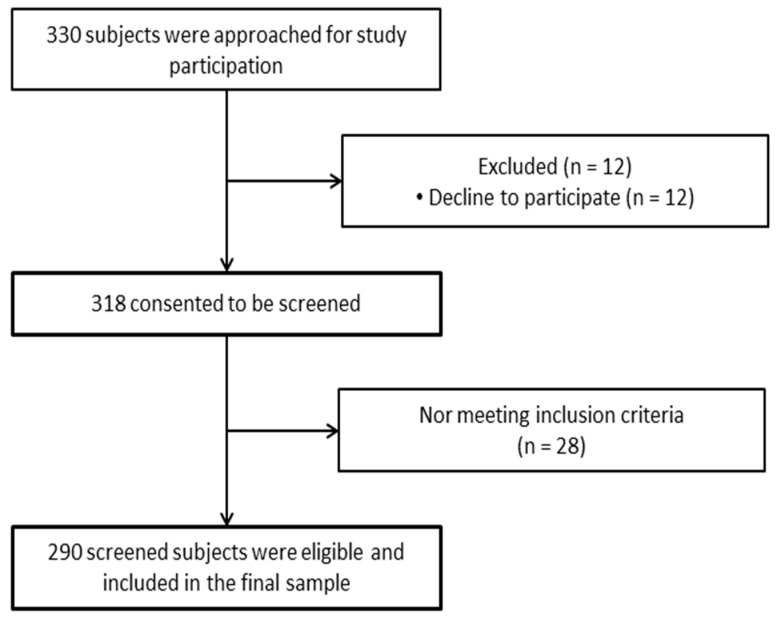
Study design flow diagram.

This was an analytical cross-sectional study, in which 330 participants were initially contacted, of which a total of 290 participated in the study (Figure 1). Participants were recruited through various municipal sports centers in Manilva, Pizarra, Baeza, and Álora. Data collection was carried out from December 2017 to March 2018. The inclusion criteria for this study were: being adults over 60 years of age living in the community, being able to complete all the questionnaires, and signing a written informed consent form. Exclusion criteria were: having any condition that contraindicates the performance of physical tests, diseases that could cause alterations in balance or functional activity, and neuropsychiatric alterations with the potential to influence the answers to the questionnaires. This study was approved by the Ethics Committee of the University of Jaén (DEC.17/5.TES) and was carried out with respect to the guidelines of the Declaration of Helsinki. To calculate the sample size, at least 20 subjects were required for each of the events in the multivariate linear regression model [23]. In this study, 13 possible events were used: acoustic and optical reaction time and the combination of both, functional mobility, muscle strength, sex, age, occupation, educational attainment level, marital status, BMI, and smoking habits. Consequently, more than 260 participants were required. The final number of participants was 290.

### 2.2. Study Outcomes

All participants were interviewed by experts, who recorded the information.

#### 2.2.1. Sociodemographic and Anthropometric Data

Sociodemographic data such as sex, age, occupation, educational attainment level, civil status, and smoking habits were collected. Weight was measured with a 100 g–130 kg precision digital weight scale (Tefal), and height was measured using an Asimed T201-T4 adult height scale. Body mass index (BMI) was measured by dividing the individual’s weight (kg) by their height squared (m^2^). A BMI ≥30 kg/m^2^ is indicated.

#### 2.2.2. Gait Speed

Reaction times were measured by means of an optoelectric detection device, the OptoGait reaction time reactivity system (Microgate Italia, Bolzano-Bozen, Italy). The transmitter bar has 96 light-emitting diodes (LEDs) that communicate in the infrared spectrum. In the front is the receiver bar, which has the same number of LEDs. The bars of both the OptoGait transmitter and receiver were positioned opposite each other on the floor to leave enough room to place both feet inside. Reaction times of the lower extremities were evaluated both acoustically (ALLRT), with the stimulus being a pre-recorded sound signal, and visually (OLLRT), with the visual stimulus being a change in color on a computer screen, a change in the light from an external source, or a combination of both. In optoacoustic reaction measurement (OALLRT) the stimulus is not specified to be visual or acoustic and the participant must react to any or both stimuli. In all conditions, participants were asked to lift their foot when they received the stimulus, taking that foot out of the area while the other acts as a pivot. Two training attempts and three experimental tests were carried out, with one minute of rest between tests.

#### 2.2.3. Functional Mobility

Functional mobility was measured through the timed up-and-go test (TUG), frequently used in older people living in the community [24]. This test requires getting up from a chair, walking three meters, turning around, and sitting back in the chair [25]. The time required by the individual to complete the task was recorded. Each participant performed it twice, and the best time was kept.

#### 2.2.4. Handgrip Strength

Muscle strength was assessed using a dynamometer (TKK 5001, Grip-A, Takei, Tokyo, Japan). Participants were asked to exert their maximum grip strength with their dominant hand three times, with a 30 s rest between tries. For a correct grip, the dynamometer was adjusted at 5.5 size to males, and for females, the optimal grip was adjusted according to the size of the hand [26]. Low muscle strength was specified as pressure force values below 16 kg in women and 27 kg in men [7].

### 2.3. Data Analysis

Continuous variables were summarized as means and standard deviations, and categorical variables as percentages and frequencies. The Kolmogorov–Smirnov test was employed to evaluate the normal distribution of all variables. A bivariate correlation analysis was applied to evaluate the possible individual ways in which independent variables such as functional mobility and grip strength, as well as other covariates such as BMI, sex, age, and educational attainment level, are associated with optic lower-limb reaction time, acoustic lower-limb reaction time, and optic/acoustic lower-limb reaction time. In order to examine possible independent associations between study variables, both a multivariate linear regression model and a step-by-step method were employed to introduce variables into the model. Optical lower-limb reaction time, acoustic lower-limb reaction time, and optic/acoustic lower-limb reaction time were registered individually as dependent variables in separate models (significant in bivariate correlation “*p* < 0.05”) and were incorporated in multivariate linear regression. Adjusted R^2^ was utilized to calculate the effect size coefficient of multiple determination in linear models. If <0.02, R^2^ was deemed to be negligible, medium if between 0.02 and 0.15, and large if >0.35 as large. A confidence level of 95% was used (*p* < 0.05). For the association between functional mobility and grip strength with optical lower-limb reaction time, acoustic lower-limb reaction time, and optic/acoustic lower-limb reaction time after adjusting for potential confounders (i.e., age, sex, body mass index and educational attainment level). The SPSS statistical package for Windows (SPSS Inc., Chicago, IL, USA) was employed for data analysis.

## 3. Results

Table 1 displays the descriptive data of the participants. A total of 290 elderly individuals (69.35 ± 5.55 years) finally took part in the study. Most of the participants were married or living with a partner (63.79%), had primary education or less (84.02%), and had a mean BMI of 29.99 ± 3.91 kg/m^2^. The descriptive data of the variables analyzed in this study showed that the time for the TUG test was 8.21 ± 2.24 s. Regarding handgrip strength values were 25.33 ± 10.38 kg. Regarding the dependent variables, the optoacoustic lower-limb time reaction, optic lower-limb time reaction, and acoustic lower-limb time reaction were 0.70 ± 0.26 s, 0.67 ± 0.20 s, and 0.68 ± 0.21 s, respectively.

The bivariate analysis (Table 2) showed that all the dependent variables analyzed in the present work (i.e., optoacoustic lower-limb time reaction and acoustic lower-limb reaction time) exhibited a significant negative correlation with handgrip strength. When analyzing OALLRT, significant negative correlations were also observed with handgrip strength. Regarding longer reaction times in ALLRT, a correlation with a decrease in handgrip strength was observed. As for the covariables included in the analysis, longer reaction times in OALLRT and OLLRT were associated with lower educational attainment levels and increased age.

The multivariate linear regression analysis (Table 3) showed several independent associations with the reaction times under study. Weaker handgrip strength was associated with increased reaction times in OALLRT; and older age was associated with longer reaction times in OALLRT (R^2^ = 0.237). Additionally, lower educational attainment levels were associated with increased reaction times in OLLRT (R^2^ = 0.215). Lastly, lower handgrip strength was associated with increased reaction times in ALLRT (R^2^ = 0.199).

## 4. Discussion

The main purpose of this study was to analyze the possible associations of muscle strength (key characteristic of sarcopenia) and functional mobility, with a range of reaction times among older adults. The results of our study, which involved 290 older adults aged >60 years, point at a significant negative association between both OALLRT and ALLRT and handgrip muscle strength (*p* < 0.05). Furthermore, regarding the covariables included in the study, educational attainment level and older age were also negatively associated with both OALLRT (*p* < 0.01) and OLLRT (*p* < 0.01 and *p* < 0.05 respectively). Finally, poorer values in handgrip muscle strength, older age, and lower educational attainment levels were risk factors for longer reaction times in ALLRT, OALLRT, and OLLRT, respectively. This implies that not only the key physical characteristic that lead to sarcopenia (low muscle strength), but also some demographic characteristics may play an important role in predicting a deterioration in several types of reaction time, which in turn could also be translated into a greater impairment in the quality of life of these patients as indicated by previous investigations [7,8,9,10,11,27]. To date, although previous studies have investigated possible associations between some of these predisposing factors and some types of reaction time [25,27], to the best of our knowledge this is one of the few studies to explore the impact of muscle strength and functional mobility in the OALLRT, ALLRT, and OLLRT values of the same sample. Therefore, beyond adding to the existing literature, our paper offers a global understanding of how these factors are able to exert a negative influence on multiple types of reaction time, and consequently on the quality of life of elderly individuals.

Our OALLRT and ALLRT results show significant negative associations (*p* < 0.05) between both types of reaction time and the lower handgrip strength of our elderly participants. This is in agreement with the fact that higher muscle strength (as measured by handgrip strength) has been associated with decreased reaction time and even preservation of cognitive function [28]. In the same line, a recent study conducted in 2018, and whose purpose was to study the relationship between handgrip strength and reaction time in three different age groups, showed how handgrip strength values increased as reaction time decreased in participants between 70–75 years, indicating better general body function and health [29]. However, it must be noted that those studies did not consider lower-limb reaction time, but rather the upper limbs. Furthermore, the first of them only considered a population between 25–40 years of age. Interestingly, and despite this, results from the study conducted by Wiśniowska-Szurlej et al. in 2019 [30] highlighted how, regardless of gender, handgrip strength is associated with decreased mobility, reduced lower-limb strength, and poorer dynamic balance in elderly people (65–85 years). Hence, taking into account that low handgrip strength is enough to start an intervention 7], that handgrip strength is an overall measurement of body strength in older adults [31,32,33] and considering that handgrip strength is not only associated with reduced lower-limb strength [31], but also with higher upper-limb reaction times [28,29], we dare speculate that all these facts taken together could explain the significant negative association between handgrip strength and both OALLRT and ALLRT in our elderly participants.

In addition to handgrip strength, our results suggest that some demographic characteristics such as lower educational attainment level and older age seem to have a negative influence over both OALLRT and OLLRT, which can be translated into higher reaction times. These findings confirm the results of previous studies that have already highlighted the impact of education and age on reaction time [34,35]. Concretely, Tun and Lachman suggested, in a study with a sample of 3616 participants aged 32–85 years, that advanced educational attainment levels were associated with decreased reaction times in elderly people compared to less-educated individuals [34]. Similarly, several studies have shown how reaction times to simple sensory stimuli (auditory or light flashes) are known to increase with age [36,37]. However, in our study the tasks employed to assess reaction times differ from those in the literature, as the majority of them measure upper-limb reaction times rather than those of the lower limbs. For all of these reasons, the impact of handgrip strength, as well as the impact of education and age over the reaction time of the lower limbs, should be confirmed and explored in greater detail in future studies involving elderly populations. Likewise, we expected to find significant negative associations between functional mobility (measured through TUG) and reaction times, given that better physical performance (again, through TUG) has been previously linked to improved handgrip strength and aerobic capacity (6 min walk test), balance (one-leg stand), and mobility (usual and maximal walking speeds) [38]. At this point, one possible explanation for the fact that our study failed to find significant differences among our participants is that the sample in that study is twice the size of ours.

Finally, the multivariate regression analysis showed associations for some predisposing factors and various types of lower-limb reaction times. OALLRT and ALLRT were both found to be negatively affected by a lower handgrip strength and, even independently, OALLRT was found to be negatively impacted by older age. Lastly, OLLRT was found to be negatively impacted by lower educational attainment levels. Henceforth, health professionals should consider that not only physical factors, but also demographic characteristics, may play an important role in both general function mobility and quality of life in older adults.

The main limitation of our study is due to its cross-sectional design, which did not allow for determining casual links between muscle strength and functional mobility, and several types of reaction time. Secondly, there is the huge difference between men and women. Lastly, muscle quantity and quality-related outcomes have not been considered, which could represent a relevant gap. However, although bioimpedance analysis is one of the most common use methods to assess these variables, it must be remarked that body composition parameters are instrument-dependent and that instrumental sensitivities could be different when implementing different technologies [39]. Therefore, the results presented in this study should be interpreted with caution. Despite the aforementioned weakness, this study not only assessed predisposing factors of sarcopenia through validated instruments that have been successfully implemented in previous research [20,21], but also established possible associations with several types of lower-limb reaction times, which could be translated into serious impairments on the quality of life of older adults.

## 5. Conclusions

In conclusion, the findings of the present study suggest that among people aged >60 years the risk factors for sarcopenia, specifically muscle strength, were independently associated with lower-limb reaction times. Specifically, our results show an association between increased muscle strength and decrease reaction times in OALLRT. Furthermore, increased muscle strength was associated with reduced reaction times in ALLRT but, contrary to our hypothesis, functional mobility did not appear to be associated with any reaction time variable. These findings allow us to suggest that gathering information about the risk factors for sarcopenia may prove useful when considering the reaction times of people aged 60 years and over.

## Figures and Tables

**Table 1:** Descriptive characteristics by all sample and split by sex (n = 290).

Characteristics.		Values Total = 290		Values	Men = 51	Values	Women = 239
Age (Years)		69.35	5.55	71.37	5.36	68.74	5.48
BMI (kg/m^2^)		29.99	3.91	30.07	3.87	29.97	3.93
Occupational status, n (%)	Retired	263	90.68	38	74.50	225	94.15
	Working	9	3.10	7	13.72	2	0.83
	Unemployed	18	6.20	6	11.78	12	5.02
Marital Status, n (%)	Single	4	1.37	2	3.92	2	0.83
	Married/cohabiting	185	63.79	14	27.45	171	71.54
	Separated/divorced/widowed	101	34.82	35	68.63	66	27.61
Educational status, n (%)	No formal education	80	33.10	15	29.42	65	27.19
	Primary education	154	50.92	28	54.90	126	52.71
	Secondary education	40	15.86	4	7.84	36	15.06
	University	16	7.58	4	7.84	12	5.02
Smoker, n (%)	Yes	18	6.20	12	23,52	6	2.50
	No	272	93.79	39	76,48	233	97.50
OALLRT (s)		0.70	0.26	0.72	0.30	0.69	0.24
OLLRT (s)		0.67	0.20	0.66	0.17	0.67	0.20
ALLRT (s)		0.68	0.21	0.71	0.71	0.67	0.20
TUG test (s)		8.21	2.24	7.96	2.63	8.29	2.10
Handgrip strength (kg)		25.33	10.38	35.06	11.13	22.16	7.87

BMI: body mass index; OALLRT: optoacoustic lower limb reaction time; OLLRT: optic lower limb reaction time; ALLRT: acoustic lower limb reaction time; TUG: timed up and go test.

**Table 2:** Pearson’s correlations between analyzed in this study.

	OALLRT (s)	OLLRT (s)	ALLRT (s)
TUG	0.104	0.091	0.030
Handgrip Strength	−0.172 ^1^	−0.071	−0.140 ^1^
Sex	−0.049	0.005	−0.084
Educational Status	−1.200 ^2^	−1.340 ^2^	−0.150
Age (years)	0.155 ^2^	0.148 ^1^	0.077
BMI (kg/m^2^)	−0.087	0.073	0.001

TUG: timed up and go test; BMI: body mass index; OALLRT: optoacoustic lower limb reaction time; OLLRT: optic lower limb reaction time; ALLRT: acoustic lower limb reaction time. ^1^ *p* < 0.05. ^2^ *p* < 0.01.

**Table 3:** Multivariate linear regression analyses for factors associated with time reaction parameters.

Variable		B	β	t	95% CI		*p*-Value
OALLRT (s)	Age	0.007	0.105	1.807	−0.01	0.012	0.021
	Handgrip Strength	−0.006	−0.170	−2.119	−0.011	0.000	0.035
OLLRT (s)	Educational	−0.030	−1.121	−1.985	−0.059	0.000	0.048
	Sex	0.210	0.13	2.29	0.03	0.390	0.023
ALLRT (s)	Sex	−0.131	−0.253	−3.037	−0.217	−0.046	0.003
	Handgrip Strength	−0.006	−0.228	−2.750	−0.011	−0.002	0.006

B: unstandardized coefficient; β: standardized coefficient; CI: confidence interval; TUG: timed up and go test; OALLRT: optoacoustic lower limb reaction time; OLLRT: optic lower limb reaction time; ALLRT: acoustic lower limb reaction time.

## Data Availability

The data shown in this study are available upon request from the corresponding author. The data is not available to the public, since taking into account the sensitive nature of all the questions asked in this study, all participants were guaranteed that the data obtained would be confidential and would not be shared.
